# Detecting Low Frequent Loss-of-Function Alleles in Genome Wide Association Studies with Red Hair Color as Example

**DOI:** 10.1371/journal.pone.0028145

**Published:** 2011-11-29

**Authors:** Fan Liu, Maksim V. Struchalin, Kate van Duijn, Albert Hofman, André G. Uitterlinden, Cornelia van Duijn, Yurii S. Aulchenko, Manfred Kayser

**Affiliations:** 1 Department of Forensic Molecular Biology, Erasmus University Medical Center, Rotterdam, The Netherlands; 2 Department of Epidemiology, Erasmus University Medical Center, Rotterdam, The Netherlands; 3 Department of Internal Medicine, Erasmus University Medical Center, Rotterdam, The Netherlands; Emory University School Of Medicine, United States of America

## Abstract

Multiple loss-of-function (LOF) alleles at the same gene may influence a phenotype not only in the homozygote state when alleles are considered individually, but also in the compound heterozygote (CH) state. Such LOF alleles typically have low frequencies and moderate to large effects. Detecting such variants is of interest to the genetics community, and relevant statistical methods for detecting and quantifying their effects are sorely needed. We present a collapsed double heterozygosity (CDH) test to detect the presence of multiple LOF alleles at a gene. When causal SNPs are available, which may be the case in next generation genome sequencing studies, this CDH test has overwhelmingly higher power than single SNP analysis. When causal SNPs are not directly available such as in current GWA settings, we show the CDH test has higher power than standard single SNP analysis if tagging SNPs are in linkage disequilibrium with the underlying causal SNPs to at least a moderate degree (r^2^>0.1). The test is implemented for genome-wide analysis in the publically available software package GenABEL which is based on a sliding window approach. We provide the proof of principle by conducting a genome-wide CDH analysis of red hair color, a trait known to be influenced by multiple loss-of-function alleles, in a total of 7,732 Dutch individuals with hair color ascertained. The association signals at the *MC1R* gene locus from CDH were uniformly more significant than traditional GWA analyses (the most significant P for CDH = 3.11×10^−142^ vs. P for rs258322 = 1.33×10^−66^). The CDH test will contribute towards finding rare LOF variants in GWAS and sequencing studies.

## Introduction

Genome-wide association studies (GWAS) have successfully identified thousands of common variants associated with many complex human phenotypes including common diseases (www.genome.gov/gwastudies). However, with a few exceptions, common variants identified to date explain only a small fraction of the overall heritability of the traits studied. It was speculated that searching for common variants with increasingly smaller effects are unlikely to substantially account for the missing heritability [1]. Thus, there have been calls for shifting the attention from genome scans of larger samples to studies of rarer variants with larger effect [2]. In particular, it has been proposed that heterozygous loss-of-function (LOF) variants may account for an essential portion of the missing heritability [1], [3].

LOF variants represent alleles resulting in reduced or abolished protein function by disrupting not only the protein-coding genes but also any essential genetic element, including non-coding regulatory motifs. They have a variety of forms, including single-base substitutions such as nonsense SNPs or splice site disruptions and small or larger insertions/deletions that change the reading frame or remove an entire gene. These are surprisingly common in healthy individuals in that gene disruption, as a result of positive selection, can be beneficial [4], [5]. On the other hand, people at the extremes of trait distributions are more likely to carry trait-associated LOF variants [6]. LOF variants are mostly recognized by their genetic association with a variety of phenotypes largely inherited in a recessive manner. It is important to note multiple LOF variants at the same locus can act not only in the homozygote state, but also in the compound heterozygote (CH) state, where the presence of two different LOF variant alleles at the same gene, one on each homologue chromosome, influence the phenotype. In such cases, the CH state would be much more frequent than the homozygote state for any individual variant. We thus expect a power gain by taking the CH state into account in GWAS or in genome sequencing studies.

There are numerous convincing examples that multiple LOF variants in a gene collectively influence a phenotype. Some examples are HFE and hemochromatosis [7], PLA2G7 and coronary heart diseases [8], SLC22A12/SLC2A9 and renal hypouricemia [9], [10], KCNQ1 and Jervell and Lange-Nielsen syndrome [11], NCCT and Gitelmańs syndrome [12], ABCC6/ GGCX and pseudoxanthoma elasticum [13], [14], [15], TG and congenital goiter [16], SCN5A and Brugada syndrome [17], P2RX7 and inflammatory response [18], ABCA12 and congenital ichthyoses [19], TRIM32 and nephrogenic diabetes insipidus [20], WFS1 and Wolfram syndrome [21], and CLDN16 and hypomagnesaemia [22]. Through this study we use LOF variants in *MC1R* and red hair color as an example where empirical data were available. Polymorphisms leading to complete loss of function of *MC1R* are responsible for the red hair/fair skin pigmentation phenotype [23], characterized by tendency to burn and inability to tan, and has been significantly linked to the development of UV-induced skin cancer, in particular melanoma. At least 9 distinct variants in *MC1R* contribute to an increased chance of developing red hair [23], [24], [25], [26]. The relative chance for the red hair phenotype was estimated to be, in general, 15-fold greater among the individuals carrying any single variant allele, compared to non-carriers, and 170-fold higher among homozygotes or CH carriers [23]. A GWAS in 2,986 Icelanders based on the Illumina 317K chip successfully confirmed the association between red hair and variants in *MC1R* where the most significant signal was derived from a tagging SNP (rs4785763 P = 3.2×10^−56^) [26]. The authors subsequently achieved a much stronger association by additionally genotyping two nonsynonymous SNPs not assayed on this chip (rs1805007 P = 2.0×10^−142^, rs1805008 P = 4.2×10^−95^). The fact that the causal alleles have an extraordinarily large effect which is sufficiently frequent in European populations (0.142 for rs1805007 and 0.108 for rs1805008 in HapMap CEU) allowed successful detection of the genome-wide significant signals from these tagging SNPs. However, in more common situations such LOF variants may have smaller effect sizes and can occur at lower frequencies, and so be undetectable, even if they are directly observed through the next generation sequencing techniques. Relevant statistical methods for detecting and quantifying their effects are sorely needed.

It was speculated that an increased statistical power may be achieved by analyzing multiple neighboring low-frequency variants simultaneously. Several methods have been proposed for analyzing a collection of selected rare mutations to test for group-wise association with a disease status. Recent developments in this area include the cohort allelic sums test (CAST) [27], the combined multivariate and collapsing (CMC) method [28], and the weighted sum statistic (WSS) [29]. In the CAST method, the overall frequency of all exonic alleles in a gene is compared between cases and controls. In the CMC method, all selected rare variants are collapsed and treated as a single common variant allele. The WSS method jointly analyzes a group of rare mutations to test for an excess of mutations in cases. Madsen et al. [29] compared the performance of CAST, CMC, and WSS and showed that WSS was the most powerful under four genetic models. In general, the power of these methods depends on the portion and the frequency of causal variants included. However, none of these methods focused on the CH and they are most suitable for analyzing exonic regions with a collection of rare and possibly functional alleles.

Here, we aim to develop a computationally efficient method to screen for multiple LOF variants, which does not rely on function annotation. The performance of this method is evaluated based on simulated phenotypes and real genotypes from the Illumina 550K chip available for 10,213 Dutch individuals from the Rotterdam Study, and compared with single SNP analysis and WSS. Finally, we provide a proof of principle using a GWAS of red hair in 7,732 participants who provided information on their hair color.

## Materials and Methods

### Rotterdam Study, microarray genotypes, and hair color data

The Rotterdam Study (RS1) [30] has been in operation since 1990 and initially included 7,983 participants living in Rotterdam, The Netherlands. The RS2 [31] is an extension of the cohort, started in 1999 and includes 3,011 participants. The RS3 [32] is a further extension of the cohort started in 2006 and includes 3,932 participants. RS1 and RS2 were genotyped using the Infinium II HumanHap550 K Genotyping BeadChip version 3.and RS3 was genotyped using Human 610 Quad Arrays of Illumina. Collection and purification of DNA, genotyping, imputation, merging, and quality control details have been described before [33], [34]. Hair color was collected in RS1 and RS2 by means of a questionnaire, with self reporting of 4 hair color categories; fair, brown, red, or black when young. After quality control, this study included a total of 10,213 individuals with 550 K genotyped SNPs, among whom 7,732 individuals provided hair color information (N red hair = 241). The Medical Ethics Committee of Erasmus Medical Center, Rotterdam, approved this study. All participants provided written informed consent.

### *MC1R* SNP genotyping

Multiple LOF mutations in *MC1R* cause red hair color. These mutations are largely recessive when considered individually and interact with each other in compound heterozygotes. Two SNPs rs1805007 (R151C) and rs1805008 (R160W) known to have the largest effects [24] but not present on the Illumina 550 K chip, were genotyped separately using melt curve genotyping. The assay design and primer synthesis were done by Tib Molbiol (Berlin, Germany, **Table S1**). For laboratory details see Text S1.

### Expected P values from the CDH test of 2 causal SNPs

The expected P values from the CDH test of 2 causal SNPs was mathematically derived as described below (also illustrated using an excel macro **Table S2**). Consider two physically close SNPs with low MAFs (1–5%). When their LD is low (as measured by *r^2^*), they approximately and independently follow HWE. The frequency of the combined genotypes is expected to follow: 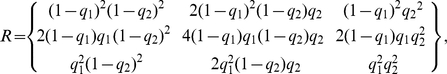
where *q_1_* and *q_2_* are the frequencies of minor alleles. Note here because *q_1_* and *q_2_* are small, *R_(2,3)_*, *R_(3,2)_*, and *R_(3,3)_* are close to zero. The CH state *R_(2,2)_* is more frequent than the homozygote state of either SNP (*R_(1,3)_* and *R_(3,1)_*), for example, when *q_1_* = *q_2_*, 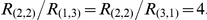



Consider a genetic model in which the homozygote and compound heterozygote genotypes lead to an increased prevalence of a binary phenotype, so that the joint penetrance table of the two SNPs can be modeled using a baseline prevalence α, together with a *GRR*, denoted as *β* here.
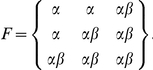



Given the total sample size *n*, the expected genotype count in cases is the element by element multiplication of *R* and *F*

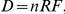
 as well as in controls
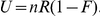



Note here we consider population based studies typically consist of a large number of healthy individuals and a small number of cases in terms of rare diseases or extreme phenotypes. This is different from the conventional case-control designs where subjects are selected based on the status of a particular disease. Therefore, *n* needs to be sufficiently large to reach reasonable power, for example, one would need 10,000 population samples to obtain 500 cases for a phenotype with 5% prevalence. However, the definitions of *D* and *U* can be easily modified if the number of cases and controls are fixed by design.

Based on *F*, a two-by-two contingency table can be formed by collapsing the lower triangle cells in both cases and controls
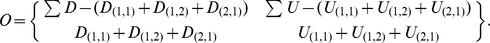



In single SNP analysis, a two-by-three table can be formed,
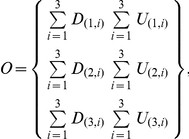
where *O* is an expected matrix of counts under the alternative hypothesis (not confused with real observations). The Chi-square value is computed using standard operations for contingency tables,
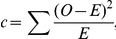
which follows the Chi-square distribution with 1 *df* for CDH test and 2 *df* for a single SNP test. The expected P values from the CDH analysis of causal SNPs are compared with those from the single SNP analysis under comparable parameters, in which we set *q* = *q_1_* = *q_2_* for illustration purposes.

### SNP sampling and trait simulation

Two physically close (<200 kb) SNPs *S1* with alleles *a* and *A* (frequency of A 1% to 5%) and *S2* with alleles *b* and *B* (frequency of B 1% to 5%) were randomly sampled 10,000 times without replacement over the Illumina 550 K chip in the Rotterdam Study (N individuals = 10,213). The *r^2^* values between SNPs *a* and *b* are derived (****). For each SNP pair, we simulated a set of binary trait status at the fixed baseline prevalence of 5% under various *GRR* ranging from 1 to 10, where *GRR_AA_* = *GRR_BB_* = *GRR_aAbB_*. The *GRR* = 1 represents the null hypothesis of no genetic association. The tagging SNP *S3* with alleles *c* and *C* is selected if it is in LD with *S1* and the tagging SNP *S4* with alleles *d* and *D* is selected if it is in LD with *S2* based on various *r^2^* thresholds (ranging from 0 to 1) without any constraint on MAF. The SNPs *S1*, *S2*, *S3*, and *S4* were tested for association with the simulated trait separately using a Chi-squared test with 2 df. The CDH test was conducted for the collapsed genotypes between SNPs *S1* and *S2* and between *S3* and *S4* using Chi-squared test with 1 df.

### Compare CDH and WSS

Madsen *et al.* [29] have compared the performance of the CAST, CMC, and WSS methods for testing associations involving rare variants and showed that WSS was the most powerful under four genetic models: recessive-set, recessive, additive and dominant. The recessive-set model is the same model as considered in this study. We compared the power of CDH with WSS under recessive-set model using simulations and focus on the scenarios whether or not causal SNPs were directly observed. Under both scenarios, the proportion of causal SNPs in a genomic region is variable and other parameters are fixed (*GRR* = 10, N individuals = 10,000, α = 0.05). WSS was implemented as described in [29] using a permutation correction of *k* = 1000 as suggested. The CDH test was conducted in a pair-wise manner and the minimal P value was Bonferroni corrected by the total number of tests (n(n−1)/2). The P-value threshold of 0.05 was used for rejecting the null hypothesis of no association. A region spanning 200 kb was randomly sampled 10,000 times over the Illumina 550 K chip. For each sampling, a binary trait was simulated by considering a portion of the low frequency variants (MAF<0.05) in the region to be causal under the recessive-set model. Other parameters were fixed (α = 0.05, *N* = 10,000, and *GRR* = 10 for carriers of any homozygote or CH genotype of the causal variants). Four scenarios were investigated where (1) all SNPs in the region were analyzed by CDH, (2) all SNPs with MAF<0.05 were analyzed by WSS, (3) all non-causal SNPs were analyzed by CDH, and (4) all non-causal variants with MAF<0.05 were analyzed by WSS.

We also compared WSS with CDH using empirical hair color data. The *MC1R* region from 87.88 to 88.69 Mb on chromosome 16 encompassed 90 genotyped SNPs with call rate>95% and was selected for testing association with red hair using CDH and WSS. The two additionally genotyped causal SNPs rs1805007 and rs1805008 were included in or excluded from the region, mimicking the scenarios where causal variants are directly available or not. SNPs in this region were cumulatively included into the WSS analysis according to their MAF in ascending order. All SNPs in the MC1R region were analyzed by CDH in a pair-wise manner, and the minimal P value was Bonferroni corrected by the total number of tests (n = 4005).

### GWA analysis

The GWA analysis was conducted using GenABEL [35] and followed closely the methods previously described [34]. The inflation factor for red hair color was 1.01. Adjusting for gender and the main principal components from the multidimensional scaling analysis did not alter GWA results. Age was adjusted at the stage of phenotype ascertainment (recalled hair color when young). Single SNP analysis was performed using a score test (qtscore) in GenABEL with 2df. In order to check if CH, rather than double heterozyotes, may indeed explain the identified association, we inferred haplotypes using the expectation maximization algorithm implemented in R library haplo.stats [36]. All SNPs in this study were annotated according to the NCBI genome-build version 36.3.

## Results

### The CH model

**Figure 1:**
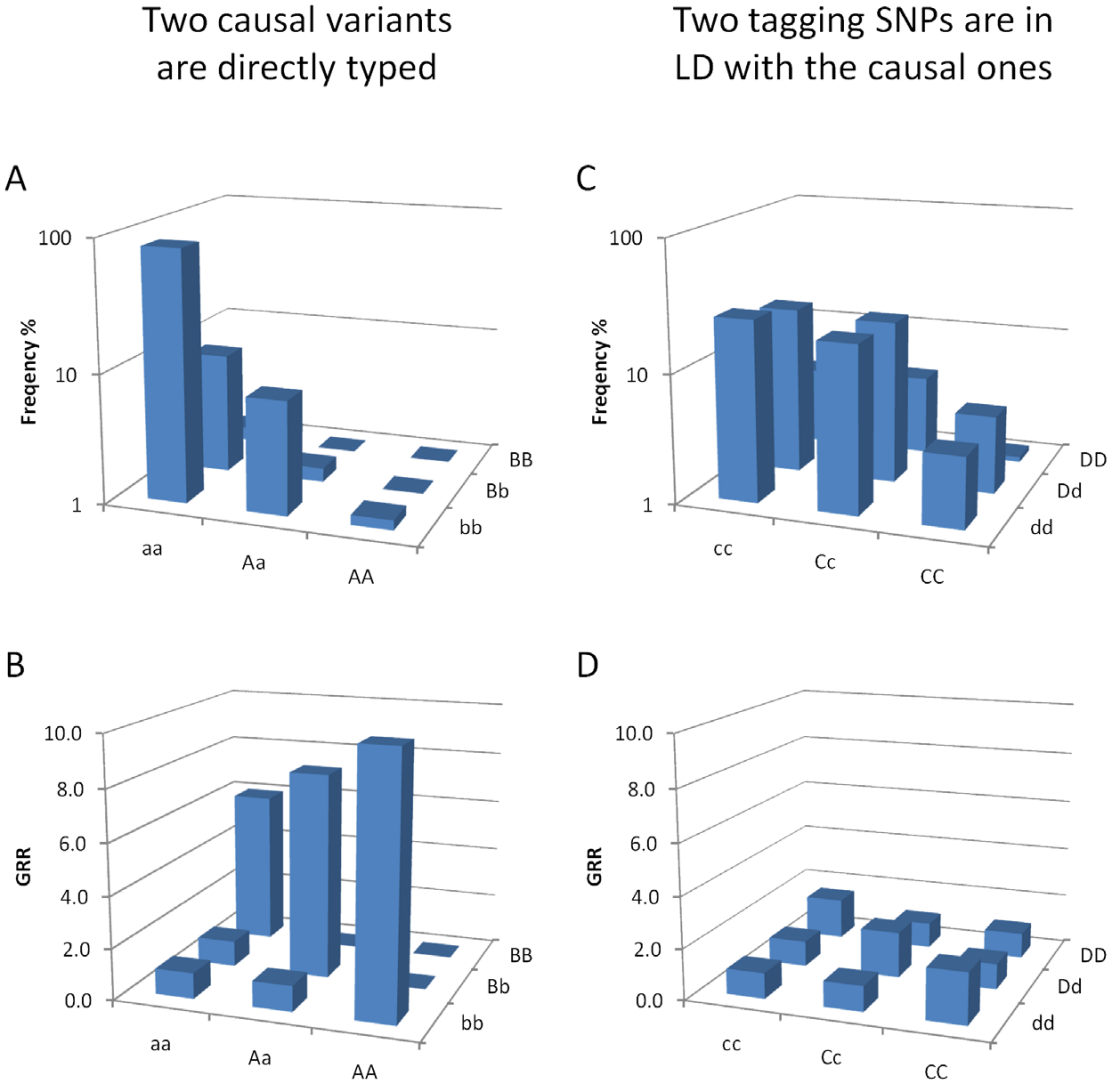
A recessive and compound heterozygote model of the phenotype. At left part of the figure (A and B) two rare recessive variants at the same gene locus are assumed to be directly genotyped. At the right part of the figure (C and D) two non-causal SNPs with higher minor allele frequencies and in LD with the causal SNPs are genotyped. The upper part of the figure depicts the logarithm scaled frequency of the cross genotypes of two variants (A and C). The lower part of the figure is an example of the genetic model under illustrative parameters. *GRR_AA_* = 8, *GRR_AaBb_* = 7, *GRR_BB_* = 6, *r_ac_^2^* = *r_bd_^2^* = 0.1 (B and D).

We consider a genetic model mimicking the situation where recessive and CH genotypes of two low-frequent variants are responsible for the genetic association with a binary phenotype (**Figure 1**). Consider two SNPs with common alleles *a* and *b* and minor alleles *A* and *B*, which are causal and of low frequency (1%<MAF<5%). Each SNP is largely recessive when considered individually, meaning homozygotes for any of the causal alleles (*AA* or *BB*) leads to an increased genotypic relative risk (*GRR*) of expressed phenotype (**Figure 1**). When two SNPs are considered jointly, not only the homozygote genotypes but also the CH genotype (*AaBb*) leads to an increased *GRR*. Here the causal alleles *A* and *B* are assumed to reside on different haplotypes as suggested previously [3], [4], [10], [11], meaning frequencies of the *AABb*, *AaBB*, and *AABB* genotypes are close to zero. We examined this assumption empirically using the *r^2^* value, because a low *r^2^* value would indicate *A* and *B* resided on different haplotypes. Note D', another frequently used LD measurement, is not necessarily low or high when *A* and *B* alleles reside on different haplotypes. A pair of two physically close (<200 kb) and low-frequent (1%<MAFs<5%) SNPs was resampled (N resampling = 10,000) over the genome (Illumina 550 K chip) in the Rotterdam Study (N individuals = 10,213). The majority (56.3%) of SNP pairs showed very low *r^2^* (<0.01, **Figure S1**). For the SNP pairs with low *r^2^*, the joint genotypes *aaBB* (on average 0.11%), *AAbb* (0.11%), and *AaBb* (0.25%) were small and the frequencies of *AABb*, *AaBB*, and *AABB* were close to zero (**Figure 1**). The frequency of the *AaBb* genotype was on average 2.27 times higher than that of the *AAbb* or *aaBB* genotypes. About 18% SNP pairs were in high LD (*r^2^*>0.9, **Figure S1**). Because the cross genotypes for two SNPs in high LD provided little or no additional information than that provided by either SNP alone (**Figure S2**), our model focuses on the low *r^2^* scenario. We developed a simple test, named the collapsed double heterozygote (CDH) test, to detect the association caused by this particular genetic model.

### CDH test of two causal SNPs

We first considered the scenario where two causal SNPs are directly genotyped, which is notably unrealistic for SNP microarray data but may be the case in next generation genome sequencing studies. The CDH test is based on the Chi-squared test as defined below. We denote the two causal SNPs as *S1* with alleles *a* and *A* and *S2* with alleles *b* and *B*. Let *D* and *U* be observed genotype counts in cases and controls and both follow a 3-by-3 matrix form,
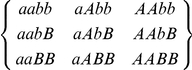



The observed matrix of counts is collapsed as, 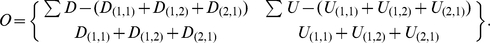



The Chi-square value is computed using standard operations for contingency tables,
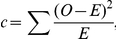
which follows the Chi-square distribution with 1 *df.* Note that there is an essential difference in the way that the genotypes are collapsed when tagging SNPs are analyzed (see the section of tagging SNPs).

**Figure 2:**
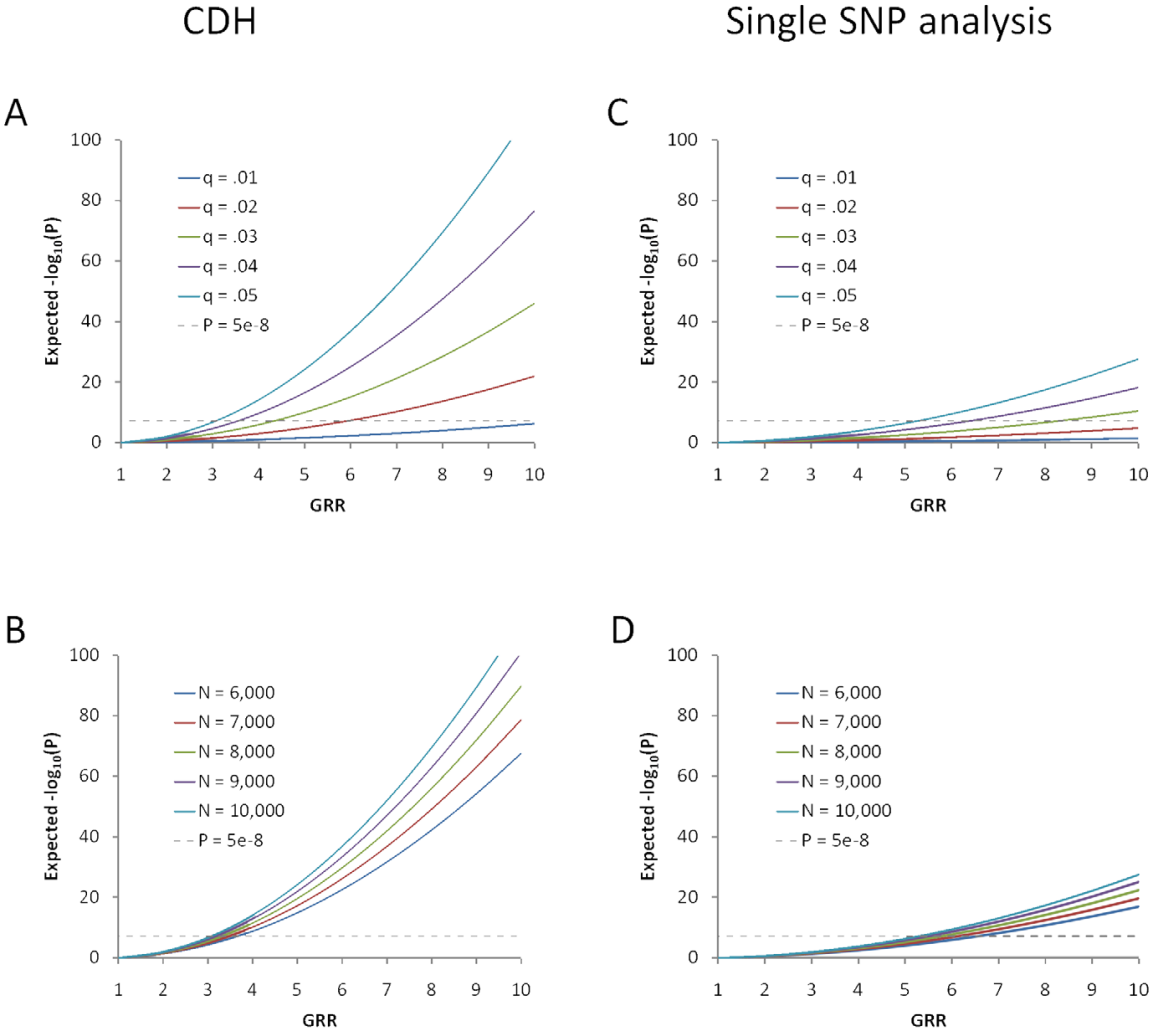
The expected P values for the CDH test. The −log_10_(P) values for two causal SNPs (on the left part of the figure, A and B) and for the single SNP chi-squared test (on the right part, C and D) are derived as a function of the genotype relative risk (*GRR_AA_* = *GRR_BB_* = *GRR_AaBb_* ranging from 1 to 10), the minor allele frequencies (*q* = *q_1_* = *q_2_* ranging from 0.01 to 0.05 when *N* is fixed at 10,000; A and C), and the total sample size *N* (ranging from 6,000 to 10,000 when *q* is fixed at 0.05; B and D). The base line prevalence of a binary phenotype is fixed at 5% in all analyses.

The expected P values from the CDH analysis of two causal SNPs and from the single SNP analysis were mathematically derived as a function of total sample size *N*, minor allele frequencies of causal SNPs *q* (*q_1_* = *q_2_* for simplicity), and *GRR* when the base line prevalence of the phenotype α is fixed at 5%. Under the CH model the CDH analysis would be expected to give more significant P values than single SNP analyses (**Figure 2**). For example, with *N* = 10,000, and 0.02<*q*<0.05, the CDH analysis is expected to give genome-wide significant P values (<5×10^−8^) for detecting reasonably large effect sizes *GRR*>3. With the same sample size, it requires higher minor frequencies (*q*≥0.05) and larger effect sizes (*GRR*>5.5) for the single SNP analyses to become genome-wide significant. Note this CDH test gives less significant P values than single SNP analysis for other genetic models where *AaBb* has no effect. For example, consider 2 independent recessive SNPs or single SNP effect (**Figure S3**). We then evaluated the type-1 error rate and the statistical power for CDH using the real genotypes from the Rotterdam Study and simulated phenotypes (**Table 1**). The type-1 error rates from CDH and the single SNP analysis, whether under the additive or recessive models, were both consistent with the expected under the null hypothesis of no association (∼5% P values smaller than 0.05). Under the alternative hypothesis (*GRR*>1), the CDH test showed much higher power than the single SNP analyses. For example, at *GRR* = 5, CDH had 52.5% power whereas single SNP analysis had less than 1% power at the significance threshold of 5×10^−8^ (**Table 1**). The gain in power using the collapsed genotypes was overwhelming even when a much more stringent threshold of 5×10^−11^ was applied only for CDH (**Table 1**). This extra adjustment allows additional multiple testing in real applications, such as genome-wide implementations based on a sliding window approach or regional implementations based on a pair-wise testing approach (see implementation section).

**Table 1:** Percentage of P values smaller than or equal to the test threshold for single SNP analysis and collapsed genotype analysis of two causal variants.

	Threshold			Threshold			Threshold
	P ≤ 0.05			P≤5e−8			P≤5e−11
*GRR*	*a*	*b*	CDH	*a*	*b*	CDH	CDH
1	5.04	4.94	4.97	0.00	0.00	0.00	0.00
2	8.13	8.04	32.98	0.00	0.00	0.46	0.10
3	14.35	15.29	67.74	0.03	0.06	8.04	2.24
4	24.57	24.02	84.58	0.07	0.15	28.34	14.54
5	35.35	34.97	92.57	0.38	0.40	52.51	35.59
6	44.99	46.06	95.80	1.17	1.00	70.58	55.59
7	54.92	55.15	97.41	2.55	2.45	81.53	71.45
8	63.18	64.05	98.57	5.11	4.86	88.62	81.48
9	69.65	70.04	99.02	8.43	8.07	92.56	87.67
10	74.72	74.95	99.36	12.86	13.07	94.81	91.69

*a*, *b*, single SNP Cochran-Armitage test of the causal variants *a* and *b.*

*GRR*, genotype relative risk. *GRR* = 1 stands for the null model of no association.

10,000 simulations for each model.

### CDH test of two tagging SNPs

**Figure 3:**
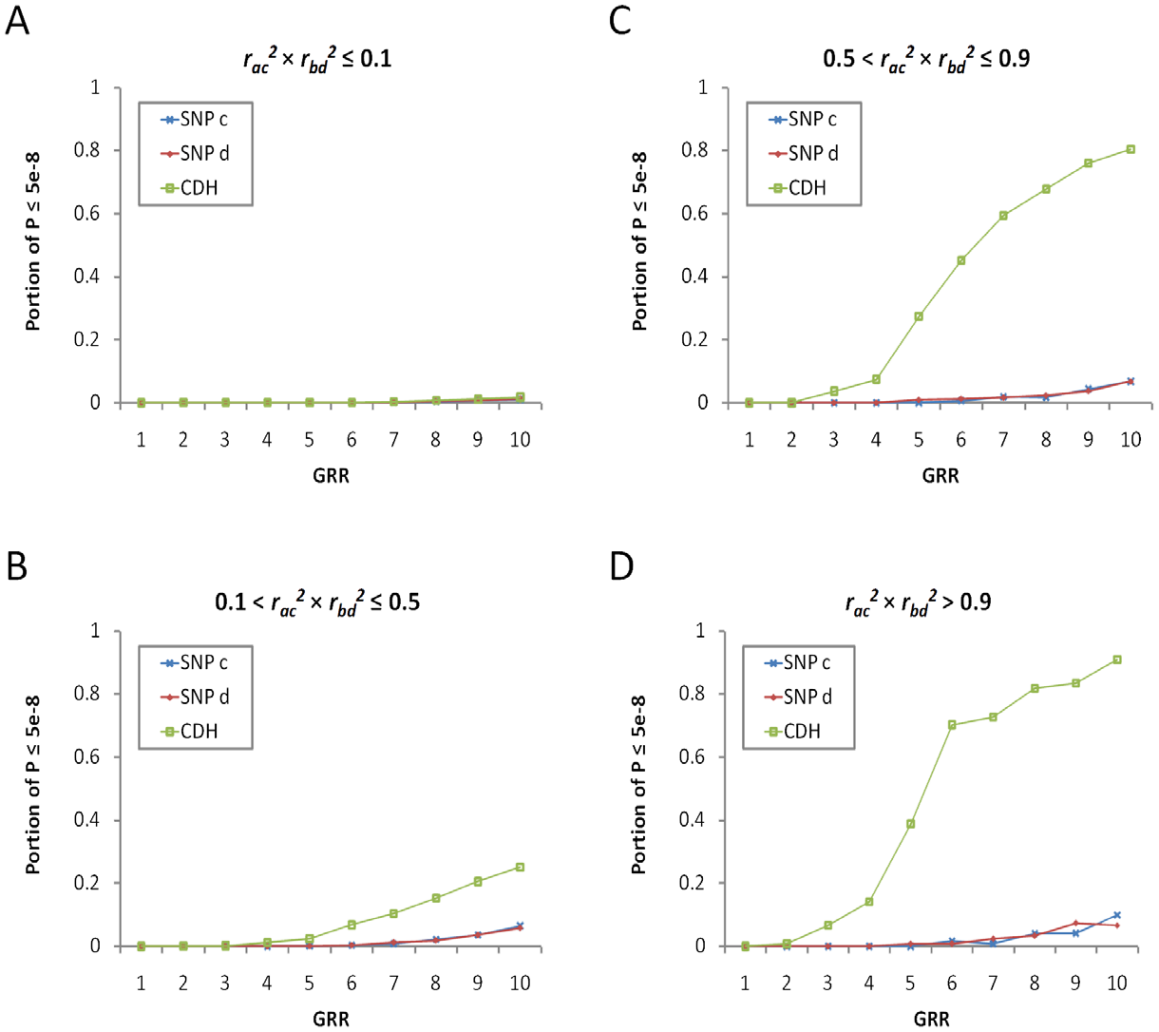
The power of CDH and single SNP analysis. Proportion of P values≤5×10^−8^ from the CDH analysis (green dots) and the single SNP Cochran-Armitage test of two tagging SNPs *c* (red dots) and *d* (blue dots). Four SNPs were re-sampled 10,000 times from the Illumina 550 K chip. SNPs *a* and *b* were physically close (<200 kb) and had low MAFs (<5%). SNP *c* was in LD with *a* and SNP *d* was in LD with *b*. The genotypic relative risk was simulated according to the genotypes of *a* and *b* under the recessive and compound heterozygote model, where *GRR_AA_* = *GRR_BB_* = *GRR_AaBb_*. The base-line prevalence of a binary phenotype was fixed at 5%. A, when *r_ac_^2^* × *r_bd_^2^*≤0.1; B, when 0.1<*r_ac_^2^*×*r_bd_^2^*≤0.5; C, when 0.5<*r_ac_^2^*×*r_bd_^2^*≤0.9, and D, when *r_ac_^2^*×*r_bd_^2^*>0.9.

A more realistic scenario in GWAS based on SNP microarrays consisting of mainly common variants is that only non-causal tagging SNPs were available. For this scenario we considered two tagging SNPs, *S3* with alleles *c* and *C* and *S4* with alleles *d* and *D*. The tagger *S3* was selected if it was in LD with *S1*, and the tagger *S4* was selected if it was in LD with *S2* based on various *r^2^* thresholds without constraints on MAF. For a given SNP with MAF <5% on the Illumina 550 K chip, there was a good chance (on average 72.74%) of obtaining at least one SNP with an *r^2^*>0.1 from its 100 neighboring SNPs. The chance of obtaining at least one SNP with *r^2^*>0.5 was much lower (on average 25.26%). The joint penetrance table for tagging SNPs showed a distinct interaction pattern differing from those previously considered for unlinked loci [37]. An important empirical finding was that only the off-diagonal cells in the cross-genotype table showed any increased *GRR*, but the *CCDd*, *CcDD*, and *CCDD* carriers did not have an increased *GRR* (**Figure 1**). This feature, which appeared to be an antagonistic interaction, can be explained by the very low frequency of the *AB* haplotypes (also see the section of the hair color analysis). This indicates the CDH test is preferred for analysis of the tagging SNPs but the *CCDd*, *CcDD*, and *CCDD* genotypes should be collapsed together with wildtypes. Again, let *D* and *U* be observed genotype counts in cases and controls,
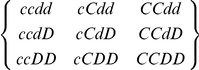
The observed matrix of counts for tagging SNP analysis is collapsed as, 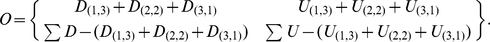
In practice this form can also be used in causal SNP analysis because *AABB*, *AAbB*, and *aABB* are negligible. Since it was difficult to mathematically derive the expected P values for the CDH test of tagging SNPs, we evaluated type-1 error and power based on simulations. The type-1 error rate for CDH test was consistent with the expected under the null hypothesis of no association (∼5% nominal P values smaller than 0.05 and 0% smaller than 5×10^−8^). Under a fixed effect size of the causal SNPs, the most important parameter for power was the *r^2^* between the causal and tagging SNPs. The product of *r_ac_^2^* and *r_bd_^2^* showed a high correlation with the test statistics of CDH (**Figure 3**). As long as *r_ac_^2^*×*r_bd_^2^*>0.1, the CDH test showed a power considerably higher than single SNP association (**Figure 3**). In particular, when *r_ac_^2^*×*r_bd_^2^*>0.5 the CDH had 27% to 91% power to detect a reasonably large effect size (*GRR*≥5) at the genome-wide significance level (P<5×10^−8^) whereas the single SNP analysis only had poor power (<10%, **Figure 3**). When *r_ac_^2^*×*r_bd_^2^* approached 1, the collapsed tagging SNPs became identical to the collapsed causal SNPs and the power of CDH reached that of the causal SNPs listed in **Table 1**. Finally, a higher power was achieved more often when MAFs of *S3* and *S4* were close to that of *S1* and *S2* as expected from the relationship between *r^2^* and MAFs.

**Figure 4:**
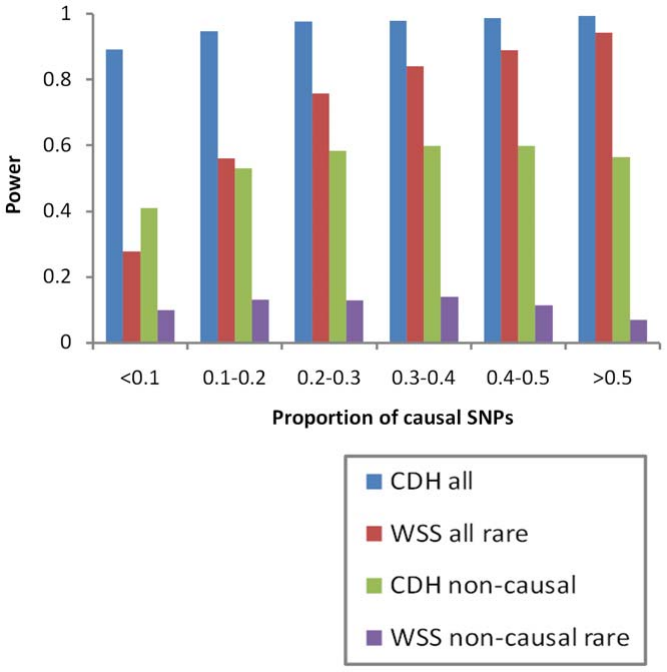
The power of CDH and WSS. The power of CDH and weighted sum statistic (WSS) [29] was plotted against the portion of causal variants in the sampled region. A region spanning 200 kb was randomly sampled 10,000 times over the Illumina 550 K chip without replacement. For each sampling, a binary trait was simulated by considering a portion of the rare variants in the region to be causal under the recessive-set model described in [29]. Other parameters were fixed (α = 0.05, n = 10,000, and *GRR* = 10 for carriers of any homozygote or CH genotype of the causal variants). Four sets of P values were derived when (1) all SNPs in the region were analyzed by CDH (blue), (2) all SNPs with MAF<0.05 were analyzed by WSS (red), (3) all non-causal SNPs were analyzed by CDH (green), and (4) all non-causal variants with MAF<0.05 were analyzed by WSS (purple). The power was defined as the portion of P values smaller than or equal to 5×10^-8^.

We further compared power of CDH with WSS through simulations. In general, the power of WSS increased when the portion of causal variants included was increased whereas CDH was much less influenced by this parameter and outperformed WSS under all scenarios investigated (**Figure 4**). The most interesting scenario is when the portion of causal variants was low (<0.1) and the causal variants were not directly observed. Under this scenario the CDH (power 0.41) clearly outperformed WSS (power 0.10).

### Software implementation

We implemented the CDH test in the software R package GenABEL [35], [38] and the core computation was implemented using external C/C++ code. The function was based on a sliding window approach and performs the CDH test for every SNP over the genome with the following *n* SNPs, which can be specified by the user. The *n* SNPs are not necessarily in or outside of known genes. The minimal P value from each slide is addressed to the first SNP of this slide and Bonferroni corrected for *n* tests. The Pearson's chi-squared or the Fisher's exact test is used depending on the number of individuals in the smallest cell. The total number of tests is *N*×*n*-*n*(*n*−1)/2, where *N* is the total number of SNPs on the genome, so for a given chip, the computational time is approximately linear to *n*. For example, with a dual core processor at 2.5 GHz, screening for 500 K SNPs in 10,000 individuals could be completed in about 7 hours for n = 100 and 14 hours for n = 200. This implementation is also practically applicable to imputed data sets and screening for 2 million SNPs could be completed in about 28 hours for n = 100 and 56 hours for n = 200. The effect of window size is relatively small as long as the SNPs cover ∼400 kb region. A window consisting of 100 SNPs is on the safe side for screening chips with 500–600 K SNPs.

### A GWAS of red hair

**Figure 5:**
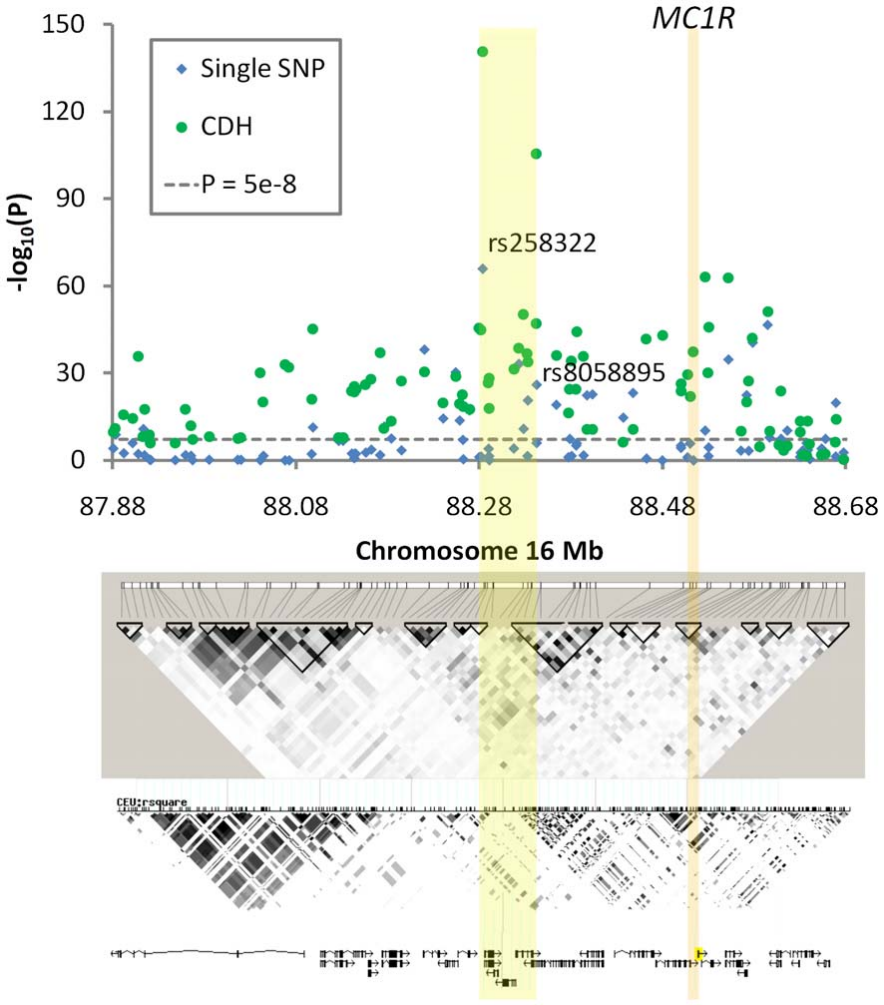
Association between SNPs at *MC1R* and the red hair color in the Rotterdam Study. The -log_10_(P) values for association with red hair color were plotted for each genotyped SNP according to its chromosomal position (blue dots) and for the CDH test in each sliding window consisting of 100 SNPs (green dots represent the left-most SNP). The LD patterns in the Rotterdam Study population and in the HapMap CEU samples (release 27) and the known genes in the region were aligned bellow according to the physical position of the SNPs (genome-build version 36.3). The orange bar indicates the physical position of the *MC1R* gene. The yellow bar indicates the region between two SNPs based on which the most significant P value of the CDH test was obtained (the left-most SNP rs258322 and the right-most SNP rs8058895).

**Figure 6:**
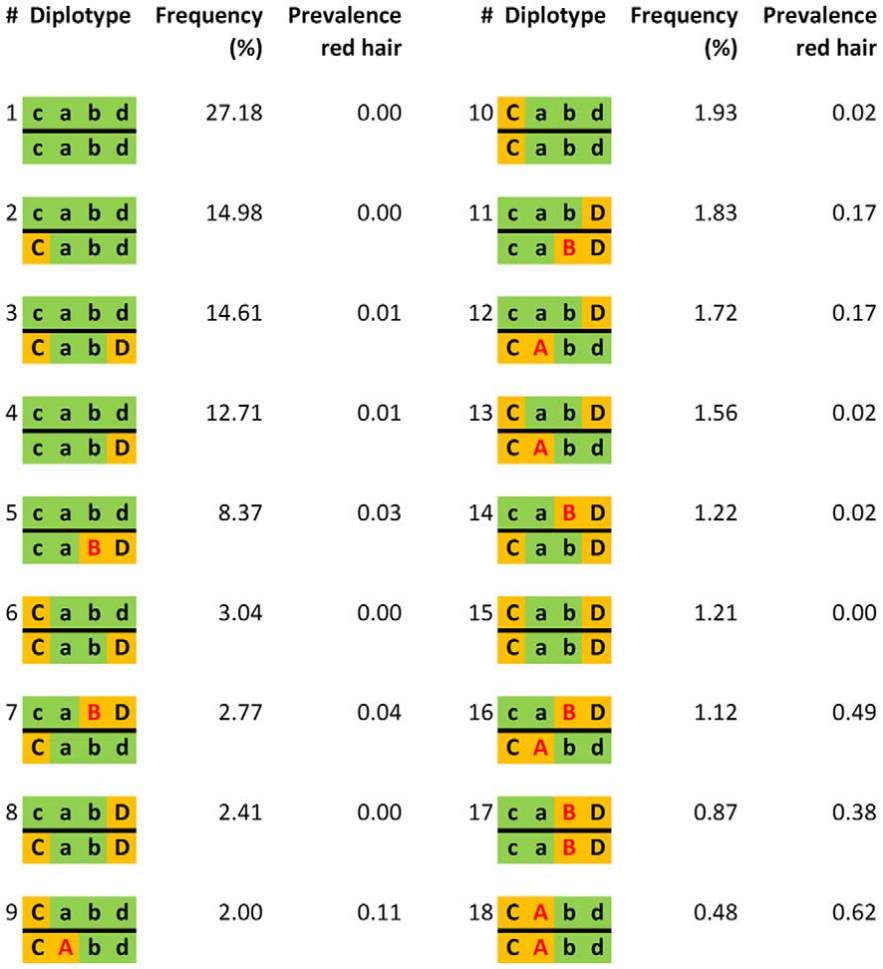
Frequency of diplotypes and the prevalence of red hair in the Rotterdam Study. The causal SNP *a* is rs1805007 and *b* is rs1805008. The tagging SNP *c* is rs2011877 and *d* is rs2302898. Causal alleles *A* and *B* are indicated in red color. Common alleles are indicated in green background and minor alleles are indicated in orange background.

We used the red hair color phenotype as the proof of principle to verify the concept that the use of collapsed genotypes is more capable of detecting the presence of multiple recessive variants at the same gene locus than traditional GWA analysis. A genome-wide CDH analysis on red-hair color was conducted in 7732 participants (N red hair = 241) of the Rotterdam Study using a window size of 100 SNPs (**Figure S4**). At chromosome 16, the 87.88 to 88.69 Mb region containing the *MC1R* gene, the association signals from the CDH analyses were uniformly higher than those from single SNP analyses (**Figure 5**). The most significant P value from CDH after the Bonferroni correction of the window size (P = 3.11×10^−142^ between SNPs rs258322 and rs8058895) was markedly more significant than seen with the single SNP association test (P for rs258322 = 1.33×10^−66^). On the other hand, there was no inflation of significant results when the hair color phenotype was randomly shuffled 100 times. Besides *MC1R,* no other region showed genome-wide significant evidence where multiple recessive variants were involved (**Figure S4**). To further illustrate the underlying mechanism that *CCDd*, *CcDD*, and *CCDD* carriers did not appear to increase *GRR*, which might be counterintuitive, we additionally genotyped two important causal SNPs for red hair [24], rs1805007 (R151C) and rs1805008 (R160W), which were not available on the original chip, in the Rotterdam Study population. Figure 6 shows diplotypes consisting of these two causal SNPs and two other tagging SNPs for *MC1R* (**Figure 6**). The causal alleles *A* and *B* represent rs1805007_T and rs1805008_T, and the tagging alleles *C* and *D* for rs2011877_C and rs2302898_T. These two tagging SNPs were selected to not be in very high LD with any causal SNPs for illustration purposes (*r_ab_^2^* = 0.007, *r_ac_^2^* = 0.147, *r_bd_^2^* = 0.216). The *CCDd* genotype is represented by diplotypes 6 and 13, *CcDD* by 8 and 14, and *CCDD* only by 15. This example empirically demonstrated the *A–B* haplotype at *MC1R* was absent in 7732 individuals. It also explained the unique “antagonistic” interaction expressed in the joint penetrance table of the two tagging SNPs (**Table 2**) where only the off-diagonal cells showed any increased prevalence of red hair. The CDH test of causal SNPs rs1805007 and rs1805008 resulted in a more significant P value (P = 4.9×10^−192^) than testing them separately (P for rs1805007 = 3.2×10^−139^, P for rs1805008 = 3.4×10^−50^). The CDH test of only tagging SNPs rs2011877 and rs2302898 also resulted in a more significant P value (P = 5.9×10^−32^) than testing them separately (P for rs2011877 = 6.8×10^−7^, P for rs2302898 = 8.9×10^−12^), confirming a power gain when multiple homozygotes and compound heterozygotes can explain the association. After significant results are obtained for the CDH test of the tagging SNPs, one can further test explicitly that CH genotypes in a collapsed set does have a different effect than the DH genotypes. This test requires diplotype information, which can be inferred statistically. In this example, we compared the red and non-red frequencies in carriers of diplotype 3 against that observed among carriers of diplotypes 7, 12, and 16 (**Figure 6**). The P value derived from this test was also highly significant (P = 3.9×10^−9^), pinpointing that CH, but not DH, could account for the identified association. Such analysis can be implemented at the genome-wide scale if the whole genome is phased. Finally, diplotypes 9, 11 and 12 seem to have intermediate prevalence compared to the recessive homozygotes or CHs. It is known that multiple causal LOF variants exist in *MC1R* and the two genotyped are the most common of these. Thus, the increased prevalence of diplotypes 9, 11 and 12 can be explained by the CH state of one of these 2 variants with another non-genotyped causal variant in *MC1R*. This also explains that an additive model (Armitage trend test) does not necessarily perform worse than an explicit recessive model in single SNP analysis when more than 2 causal recessive variants exist.

**Table 2:** Frequency of red hair phenotype as a function of genotype of two non-causal SNPs tagging the causal variants at the *MC1R* gene locus.

		rs2011877		
		GG	GT	TT
rs2302898	AA	0.00	0.02	0.14
	AG	0.02	0.06	0.01
	GG	0.22	0.01	0.00

Finally, we compared results of CDH with WSS analysis of *MC1R* region (**Figure S5**). Using the original chip without causal SNPs rs1805007 and rs1805008, the minimal P value of 1.0×10^−11^ was obtained for WSS when 7 SNPs with MAF<0.07 were included in the analysis, which is less significant than the P value from the CDH analysis of all SNP pairs in the MC1R region (Bonferroni corrected P = 7.6×10^−142^). Assuming the two causal SNPs rs1805007 and rs1805008 were available on the chip, the minimal P value (P = 2.5×10^−19^) was obtained for WSS when 14 SNPs with MAF<0.1 were included, which was also less significant than the P value obtained from the CDH analysis of all SNPs including the causal ones (Bonferroni corrected P = 1.6×10^−190^).

## Discussion

We demonstrated theoretically and empirically by simulations that using the collapsed genotypes in GWA analysis is more powerful than single SNP analysis and the WSS method in detecting the presence of multiple LOF variants at a particular gene locus. In a genome scan of the red hair color phenotype this CDH analysis resulted in considerably more significant association signals than single SNP analysis at *MC1R*. Besides *MC1R*, no other region of CH association with red hair was identified. By additional genotyping of two causal SNPs in *MC1R* we confirmed a recessive mechanism underlying this gain in statistical power. The generalizablity of CDH mainly depends on the effect sizes and frequencies of causal alleles. We expect CDH is generalizable to some of the known examples, such as HFE and hemochromatosis, where both the allele effect sizes and frequencies are comparable to MC1R alleles. Further, through simulations we showed our method is capable to find LOF alleles with smaller effect sizes (GRR>3) but not with frequencies lower than 1%. It should therefore be emphasized this approach still requires causal alleles to be at some appreciable frequency (>1%) to be effectively tested and probably not useful for exceptionally rare variants.

Here we focused on a recessive and CH model that addresses, but not restricted to, the SNP interactions caused by LOF variants. This type of SNP interaction is only a subtype of CH-like interactions, e.g. multiple gain function SNPs may well follow the CH model. However, a number of different models exist in theory, in which combinations of different variants influence a particular phenotype. A more ‘omnibus’ hypothesis-testing model may work reasonably well in multiple or most settings. Still, we believe the proposed CH model is valuable. First, it has been suggested that LOF variants are surprisingly common [4], [5] and they may account for a substantial portion of missing heritability [1], [3]. Second, the recessive model is most likely the true model underlying a significant portion of the causal variants undetected by the GWAS conducted to date. In conventional single SNP analysis, the required sample size to detect a recessive allele is a quadratic function of its frequency, which is much larger than the required sample size to detect a dominant or additive allele of the same effect size. This is regardless of the number of causal variants involved at any gene for single SNP analysis. Thus, we expect an essential portion of the currently undetected alleles to be recessive. Third, the magnitude of the power gain of this proposed model is overwhelming for detecting CH-like interactions, in particular for tagging SNP analysis. The more significant P value from the CDH test is clearly driven by the CH carriers. As also shown in the method section, when *q_1_* = *q_2_* the frequency of CH carriers is 4 times higher than homozygote carriers of single SNP, serving as the driving source of the statistical significance. Finally, CDH is computationally simple and practically applicable to large-scale data sets.

It has been repeatedly suggested [28], [29], [39], [40] that rare causal variants are likely to reside on different haplotypes. Under this scenario, the *r^2^* between two variants is small and the frequency of the *AB* haplotype is close to zero. Thus, the *AABb*, *AaBB*, and *AABB* genotype carriers are either unobservable or negligible in practice and the forming of a collapsed marker by collapsing the *AAbb*, *aaBB*, *AaBb* genotypes has been described in length previously [39], [40]. What has not been so clear is the scenario when tagging SNPs with higher minor allele frequencies are in LD with the rare causal ones, given that the frequency of the *CD* haplotype is not close to zero. Through simulations and the empirical hair color data we showed that the *CD* haplotype carriers usually do not have an increased *GRR*. By grouping the *CcDD*, *CCDd*, and *CCDD* genotype carriers together with the wild-type carriers, which is the creative element of this paper, we have shown that the tagging SNPs are capable of revealing significant signals. More importantly, iterative analysis of two tagging SNPs based on a sliding window approach is useful in genome-wide implementations. The proposed models involve only two LOF SNPs in weak LD, but of course one could envision situations in which CH effects could arise due to heterozygosity at a number of different but physically close loci, such as the *MC1R* gene exemplified here or the well-known *HLA* region. In such cases, iteratively analyzing two of the variants has an advantage over the collection-based methods [27], [28], [29] because power is not compromised by the number of unassociated SNPs included. Although the downside of this method is the additional multiple testing depending on the window size, which must be sufficiently large to cover all SNPs potentially in LD, the power gain is clearly overwhelming. For example, consider the bottom line if the whole genome is tested pair-wise in the genome-scan of red hair color, the CDH test of tagging SNPs would still result in a much more significant P value (10^−142^×10^12^≈10^−130^) than single SNP analysis (10^−66^) at *MC1R*. On the other hand, for collection-based methods [27], [28], [29], power approaches zero when more and more SNPs are included.

The use of the collapsed genotypes based on tagging or causal SNPs is conceptually distinguished. The interpretation of results may be straightforward when the causal variants are directly available as expected from full genome sequencing data. However, when they are not available and only the tagging SNPs are analyzed, i.e. based on the currently available genotyping chips, the key parameter determining the power is the strength of LD in term of *r^2^* between the underlying causal SNPs and tagging SNPs. In particular, when *r_ac_^2^*×*r_bd_^2^*>0.5 the CDH provides good to excellent power to detect a reasonably large effect size in a population based sample. A critical concern here is the portion of rare variants that are well tagged on the existing genome-wide panels. About 20% of low frequency and physically close SNP pairs from the Illumina 550 k chip have *r^2^*>0.9 (**Figure S1**), and about 25% have *r^2^*>0.5. These estimates are in line with a recent report showing panels consisting of 300–550 K SNPs capture only a small proportion of the rare non-synonymous SNPs (10–27% tagged by *r^2^*>0.5) in Europeans [41]. Thus, the portion of rare SNPs tagged by current chips is far from desirable for CDH analysis, except for some candidate traits, such as exemplified here for red hair. Reference panels such as the International HapMap Project [42] (http://snp.cshl.org/) and the 1000 Genome Project [43] have already covered up to 7.7 million newly identified rare variants in multiple human populations. The recent progress in the imputation techniques has improved the accuracy of imputing these rare variants [44]. However, in general, the imputation error rate increases as the minor allele frequency decreases across all imputation panels and genotyping chips [45]. On the other hand, using CDH to analyze the denser chips can be safely recommended for screening LOF variants, as in the Illumina 1 M chip, where the density of rare SNPs is already higher than the common ones [39], although full genome sequencing data would be ideal. Finally, regional diplotype analysis is recommended after promising regions are identified with our method. Such promising regions may be followed up by the case selection approach Wang and colleagues have proposed for deep-sequencing [40].

The chi-square statistics used here for analyzing binary traits is simple, and readily extended to general linear models for analyzing quantitative traits with or without covariates. Rather than emphasizing the advances in modern statistics, we underline the known genetic interaction between two or more LOF variants at the same gene: both homozygotes and the CH genotypes result in an increased prevalence of phenotype, and taking this into consideration increases the power in detecting them. The presence of such variants may be common and should be considered in routine analysis in genome scans, particularly for extreme phenotype designs. Our approach is useful in finding these variants in GWAS carried out with chips of ultra-high density, as well as future full genome sequencing studies.

## Supporting Information

Figure S1 **The LD r2 distribution of the physically close and rare SNP pairs on Illumina 550K chip.**
(TIF)

Figure S2 **Cross-genotypes between 2 rare SNPs in high LD.**
(TIF)

Figure S3 **Expected P values from CDH and single SNP analyses considering 2 recessive SNPs** **independently associated with phenotype.** The -log10(P) values for CDH test (A) and single SNP analysis (B and C) are plotted against the genotype relative risks of homozygote causal allele (GRR ranging from 1 to 10). Other parameters are fixed (the frequencies of causal alleles = 0.05, N = 10,000, alpha = 5%).
(TIF)

Figure S4 **Manhattan plot showing association with the red-hair color phenotype in the Rotterdam Study.** The -log10(P) values for association with red hair color are plotted for each genotyped SNP according to its chromosomal position (blue dots) and for the CDH test in each sliding window consisting of 100 SNPs (green dots).
(TIF)

Figure S5 **Association analysis of the MC1R SNPs and the red hair color using the weighted sum statistic (WSS).** All number of genotyped SNPs in the 87.88 to 88.69 Mb region of (N SNPs = 90) were included to the WSS analysis according to the minor allele frequencies in the ascending order. The −log_10_(P) values from WSS were plotted against the MAF thresholds (blue dots). The analysis was then repeated by assuming that two causal SNPs rs1805007 and rs1805008 were available on the chip (red dots). A, the −log_10_(P) values; B, the number of SNPs included in the analysis.
(TIF)

Text S1 **Laboratory details for MC1R SNP genotyping.**
(DOC)

Table S1 **Primers of two** ***MC1R*** **SNPs.**
(DOC)

Table S2 **An interactive Excel spreadsheet for illustrating the expected P values from CDH and single SNP analysis.**
(XLS)

## References

[1] Manolio TA, Collins FS, Cox NJ, Goldstein DB, Hindorff LA (2009). Finding the missing heritability of complex diseases.. Nature.

[2] Goldstein DB (2009). Common genetic variation and human traits.. N Engl J Med.

[3] Singleton AB, Hardy J, Traynor BJ, Houlden H (2010). Towards a complete resolution of the genetic architecture of disease.. Trends Genet.

[4] Sabeti PC, Schaffner SF, Fry B, Lohmueller J, Varilly P (2006). Positive natural selection in the human lineage.. Science.

[5] MacArthur DG, Tyler-Smith C (2010). Loss-of-function variants in the genomes of healthy humans.. Hum Mol Genet.

[6] Romeo S, Pennacchio LA, Fu Y, Boerwinkle E, Tybjaerg-Hansen A (2007). Population-based resequencing of ANGPTL4 uncovers variations that reduce triglycerides and increase HDL.. Nat Genet.

[7] Feder JN, Gnirke A, Thomas W, Tsuchihashi Z, Ruddy DA (1996). A novel MHC class I-like gene is mutated in patients with hereditary haemochromatosis.. Nat Genet.

[8] Song K, Nelson MR, Aponte J, Manas ES, Bacanu SA (2011). Sequencing of Lp-PLA2-encoding PLA2G7 gene in 2000 Europeans reveals several rare loss-of-function mutations..

[9] Enomoto A, Kimura H, Chairoungdua A, Shigeta Y, Jutabha P (2002). Molecular identification of a renal urate anion exchanger that regulates blood urate levels.. Nature.

[10] Matsuo H, Chiba T, Nagamori S, Nakayama A, Domoto H (2008). Mutations in glucose transporter 9 gene SLC2A9 cause renal hypouricemia.. Am J Hum Genet.

[11] Wang RR, Li N, Zhang YH, Wang LL, Teng SY (2011). Novel compound heterozygous mutations T2C and 1149insT in the KCNQ1 gene cause Jervell and Lange-Nielsen syndrome.. Int J Mol Med.

[12] Gitelman HJ, Graham JB, Welt LG (1966). A new familial disorder characterized by hypokalemia and hypomagnesemia.. Trans Assoc Am Physicians.

[13] Bergwitz C, Roslin NM, Tieder M, Loredo-Osti JC, Bastepe M (2006). SLC34A3 mutations in patients with hereditary hypophosphatemic rickets with hypercalciuria predict a key role for the sodium-phosphate cotransporter NaPi-IIc in maintaining phosphate homeostasis.. Am J Hum Genet.

[14] Lorenz-Depiereux B, Benet-Pages A, Eckstein G, Tenenbaum-Rakover Y, Wagenstaller J (2006). Hereditary hypophosphatemic rickets with hypercalciuria is caused by mutations in the sodium-phosphate cotransporter gene SLC34A3.. Am J Hum Genet.

[15] Vanakker OM, Martin L, Gheduzzi D, Leroy BP, Loeys BL (2007). Pseudoxanthoma elasticum-like phenotype with cutis laxa and multiple coagulation factor deficiency represents a separate genetic entity.. J Invest Dermatol.

[16] Ieiri T, Cochaux P, Targovnik HM, Suzuki M, Shimoda S (1991). A 3′ splice site mutation in the thyroglobulin gene responsible for congenital goiter with hypothyroidism.. J Clin Invest.

[17] Bezzina CR, Rook MB, Wilde AA (2001). Cardiac sodium channel and inherited arrhythmia syndromes.. Cardiovasc Res.

[18] Shemon AN, Sluyter R, Fernando SL, Clarke AL, Dao-Ung LP (2006). A Thr357 to Ser polymorphism in homozygous and compound heterozygous subjects causes absent or reduced P2X7 function and impairs ATP-induced mycobacterial killing by macrophages.. J Biol Chem.

[19] Akiyama M (2010). ABCA12 mutations and autosomal recessive congenital ichthyosis: a review of genotype/phenotype correlations and of pathogenetic concepts.. Hum Mutat.

[20] Borg K, Stucka R, Locke M, Melin E, Ahlberg G (2009). Intragenic deletion of TRIM32 in compound heterozygotes with sarcotubular myopathy/LGMD2H.. Hum Mutat.

[21] Hong J, Zhang YW, Zhang HJ, Jia HY, Zhang Y (2009). The novel compound heterozygous mutations, V434del and W666X, in WFS1 gene causing the Wolfram syndrome in a Chinese family.. Endocrine.

[22] Hampson G, Konrad MA, Scoble J (2008). Familial hypomagnesaemia with hypercalciuria and nephrocalcinosis (FHHNC): compound heterozygous mutation in the claudin 16 (CLDN16) gene.. BMC Nephrol.

[23] Valverde P, Healy E, Jackson I, Rees JL, Thody AJ (1995). Variants of the melanocyte-stimulating hormone receptor gene are associated with red hair and fair skin in humans.. Nat Genet.

[24] Box NF, Wyeth JR, O'Gorman LE, Martin NG, Sturm RA (1997). Characterization of melanocyte stimulating hormone receptor variant alleles in twins with red hair.. Hum Mol Genet.

[25] Beaumont KA, Shekar SN, Cook AL, Duffy DL, Sturm RA (2008). Red hair is the null phenotype of MC1R.. Hum Mutat.

[26] Sulem P, Gudbjartsson DF, Stacey SN, Helgason A, Rafnar T (2007). Genetic determinants of hair, eye and skin pigmentation in Europeans.. Nat Genet.

[27] Morgenthaler S, Thilly WG (2007). A strategy to discover genes that carry multi-allelic or mono-allelic risk for common diseases: a cohort allelic sums test (CAST).. Mutat Res.

[28] Li B, Leal SM (2008). Methods for detecting associations with rare variants for common diseases: application to analysis of sequence data.. Am J Hum Genet.

[29] Madsen BE, Browning SR (2009). A groupwise association test for rare mutations using a weighted sum statistic.. PLoS Genet.

[30] Hofman A, Grobbee DE, de Jong PT, van den Ouweland FA (1991). Determinants of disease and disability in the elderly: the Rotterdam Elderly Study.. Eur J Epidemiol.

[31] Hofman A, Breteler MM, van Duijn CM, Krestin GP, Pols HA (2007). The Rotterdam Study: objectives and design update.. Eur J Epidemiol.

[32] Hofman A, Breteler MM, van Duijn CM, Janssen HL, Krestin GP (2009). The Rotterdam Study: 2010 objectives and design update..

[33] Estrada K, Krawczak M, Schreiber S, van Duijn K, Stolk L (2009). A genome-wide association study of northwestern Europeans involves the C-type natriuretic peptide signaling pathway in the etiology of human height variation.. Hum Mol Genet.

[34] Liu F, Wollstein A, Hysi PG, Ankra-Badu GA, Spector TD (2010). Digital quantification of human eye color highlights genetic association of three new loci.. PLoS Genet.

[35] Aulchenko YS, Ripke S, Isaacs A, van Duijn CM (2007). GenABEL: an R library for genome-wide association analysis.. Bioinformatics.

[36] Schaid DJ, Rowland CM, Tines DE, Jacobson RM, Poland GA (2002). Score tests for association between traits and haplotypes when linkage phase is ambiguous.. Am J Hum Genet.

[37] Marchini J, Donnelly P, Cardon LR (2005). Genome-wide strategies for detecting multiple loci that influence complex diseases.. Nat Genet.

[38] Aulchenko YS, Struchalin MV, van Duijn CM (2010). ProbABEL package for genome-wide association analysis of imputed data.. BMC Bioinformatics.

[39] Dickson SP, Wang K, Krantz I, Hakonarson H, Goldstein DB (2010). Rare variants create synthetic genome-wide associations.. PLoS Biol.

[40] Wang K, Dickson SP, Stolle CA, Krantz ID, Goldstein DB (2010). Interpretation of association signals and identification of causal variants from genome-wide association studies.. Am J Hum Genet.

[41] Evans DM, Barrett JC, Cardon LR (2008). To what extent do scans of non-synonymous SNPs complement denser genome-wide association studies?. Eur J Hum Genet.

[42] International-HapMap-Consortium (2003). The International HapMap Project.. Nature.

[43] Via M, Gignoux C, Burchard EG (2010). The 1000 Genomes Project: new opportunities for research and social challenges.. Genome Med.

[44] Howie BN, Donnelly P, Marchini J (2009). A flexible and accurate genotype imputation method for the next generation of genome-wide association studies.. PLoS Genet.

[45] Marchini J, Howie B (2010). Genotype imputation for genome-wide association studies.. Nat Rev Genet.

