# Supplementary figures and images for: Detecting Low Frequent Loss-of-Function Alleles in Genome Wide Association Studies with Red Hair Color as Example
Source: PLoS One. 2011 Nov 29;6(11):e28145. doi: 10.1371/journal.pone.0028145 (PMC3226656; doi:10.1371/journal.pone.0028145)

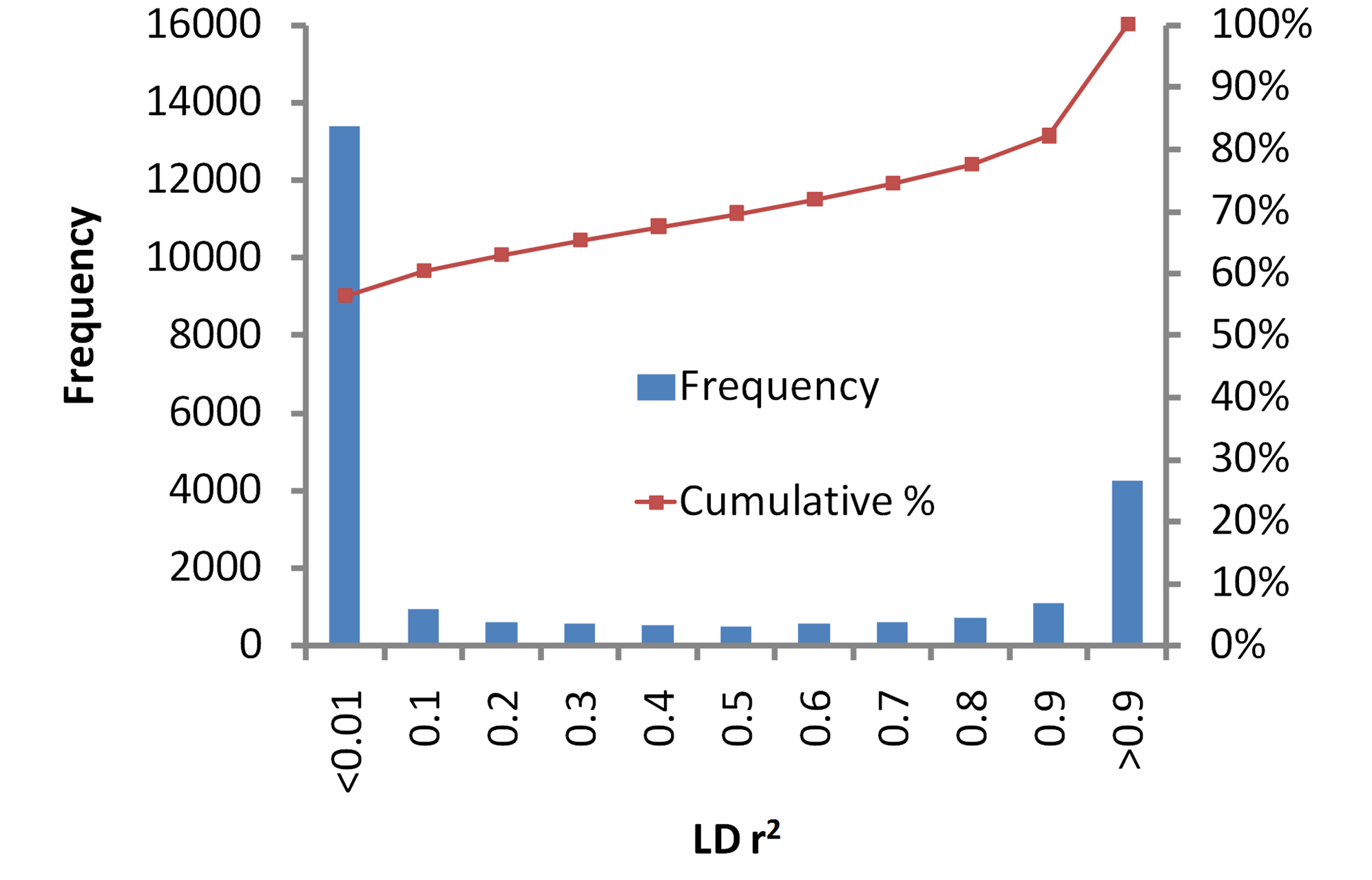

Supplement: Figure S1 — The LD r2 distribution of the physically close and rare SNP pairs on Illumina 550K chip. (TIF) [file pone.0028145.s001.tif]

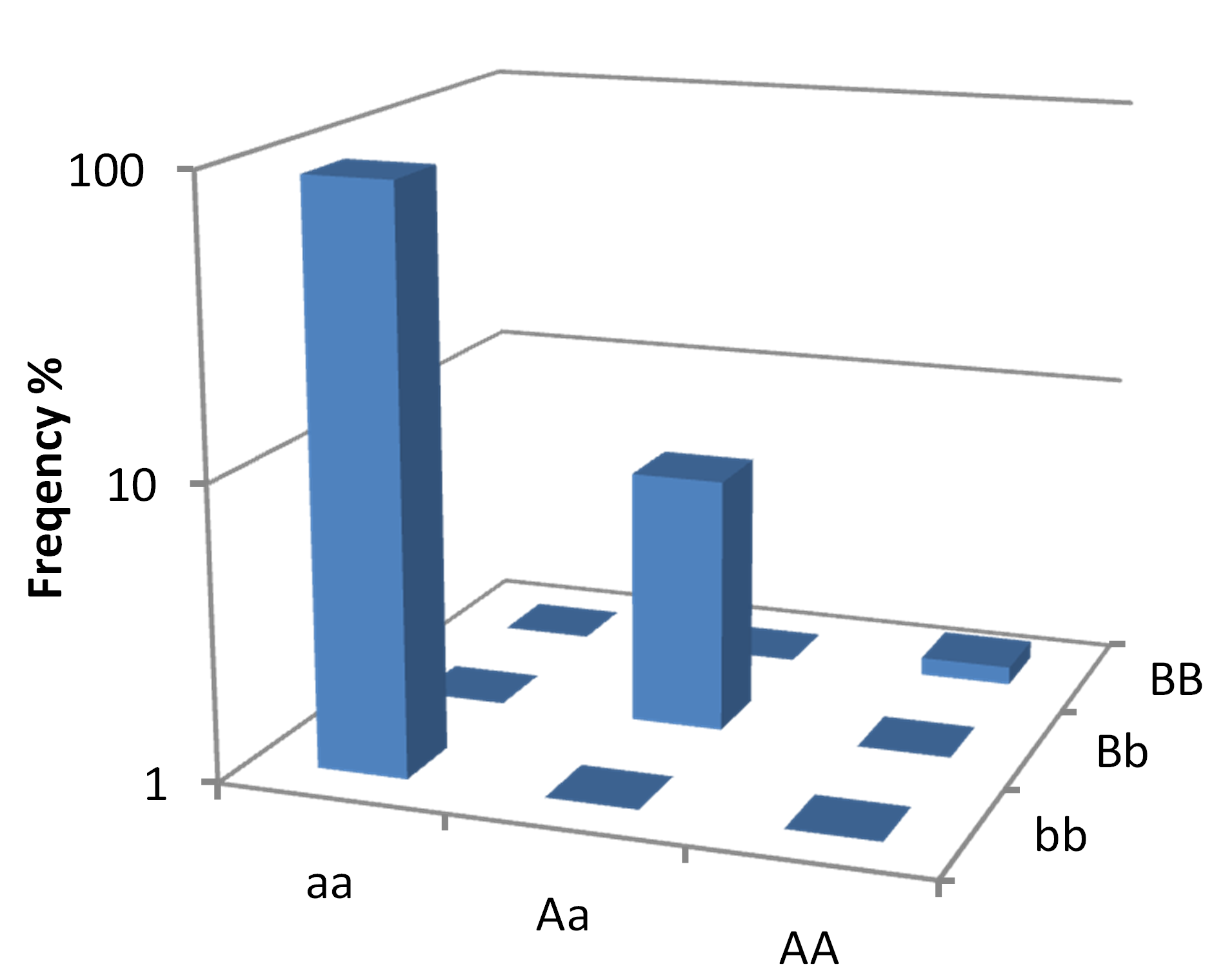

Supplement: Figure S2 — Cross-genotypes between 2 rare SNPs in high LD. (TIF) [file pone.0028145.s002.tif]

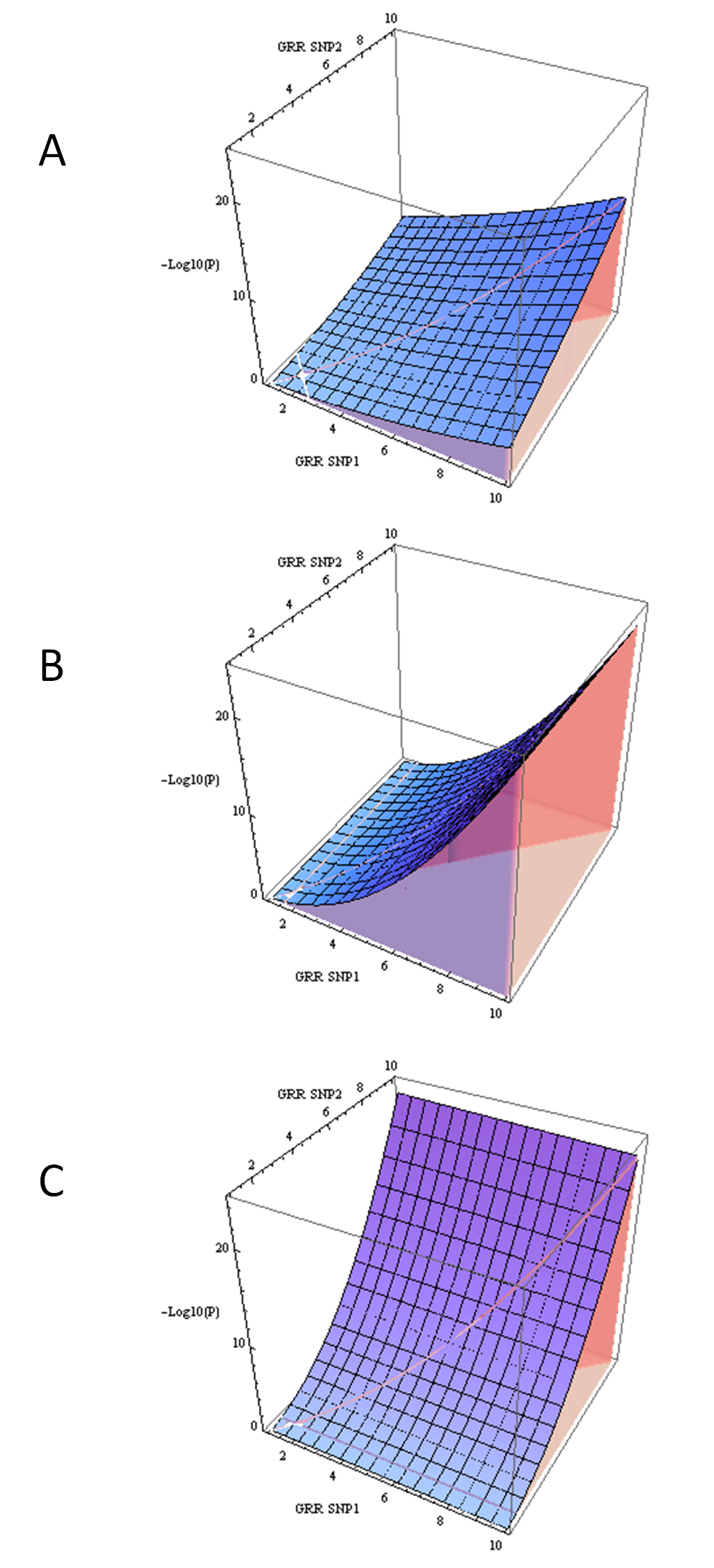

Supplement: Figure S3 — Expected P values from CDH and single SNP analyses considering 2 recessive SNPs independently associated with phenotype. The -log10(P) values for CDH test (A) and single SNP analysis (B and C) are plotted against the genotype relative risks of homozygote causal allele (GRR ranging from 1 to 10). Other parameters are fixed (the frequencies of causal alleles = 0.05, N = 10,000, alpha = 5%). (TIF) [file pone.0028145.s003.tif]

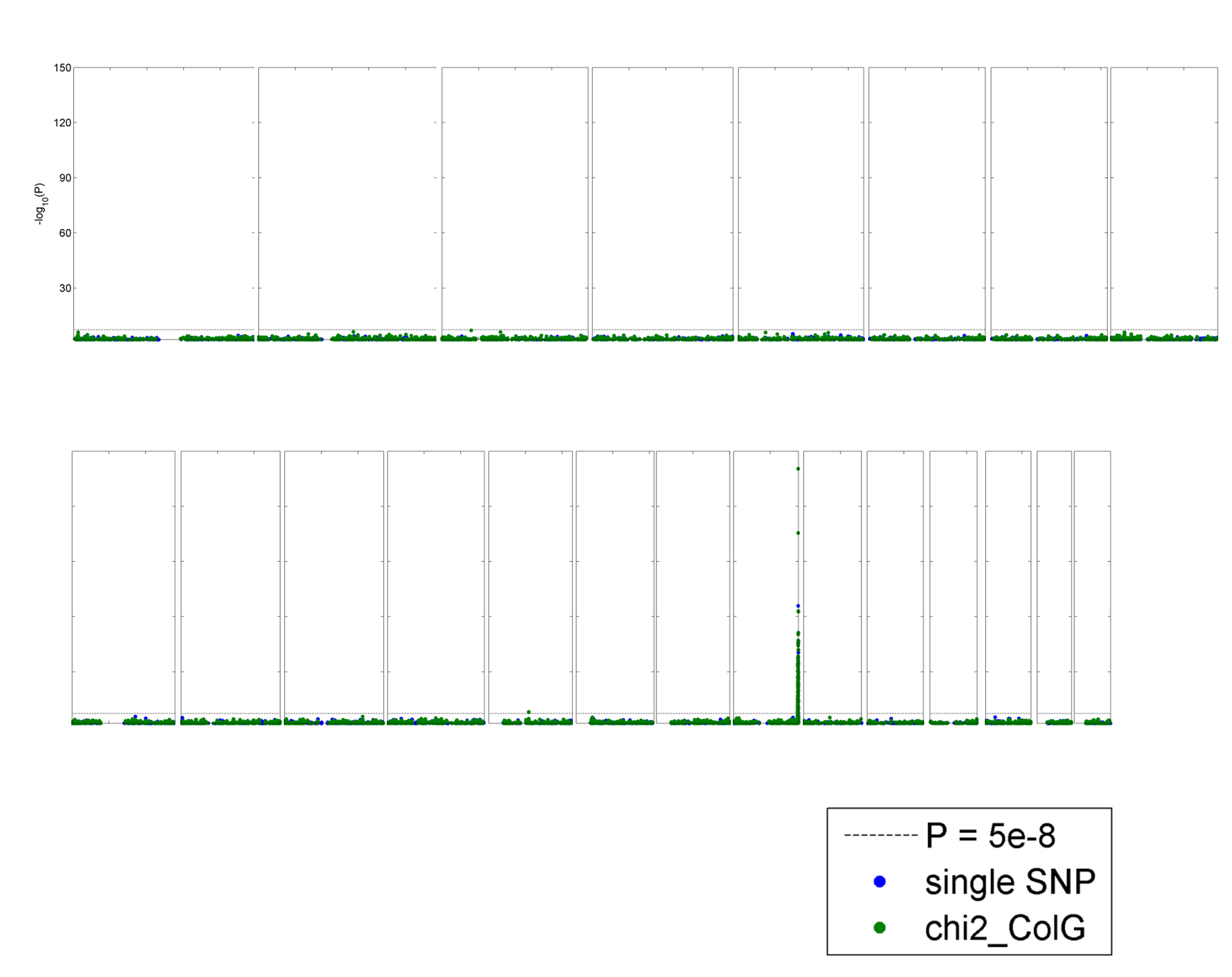

Supplement: Figure S4 — Manhattan plot showing association with the red-hair color phenotype in the Rotterdam Study. The -log10(P) values for association with red hair color are plotted for each genotyped SNP according to its chromosomal position (blue dots) and for the CDH test in each sliding window consisting of 100 SNPs (green dots). (TIF) [file pone.0028145.s004.tif]

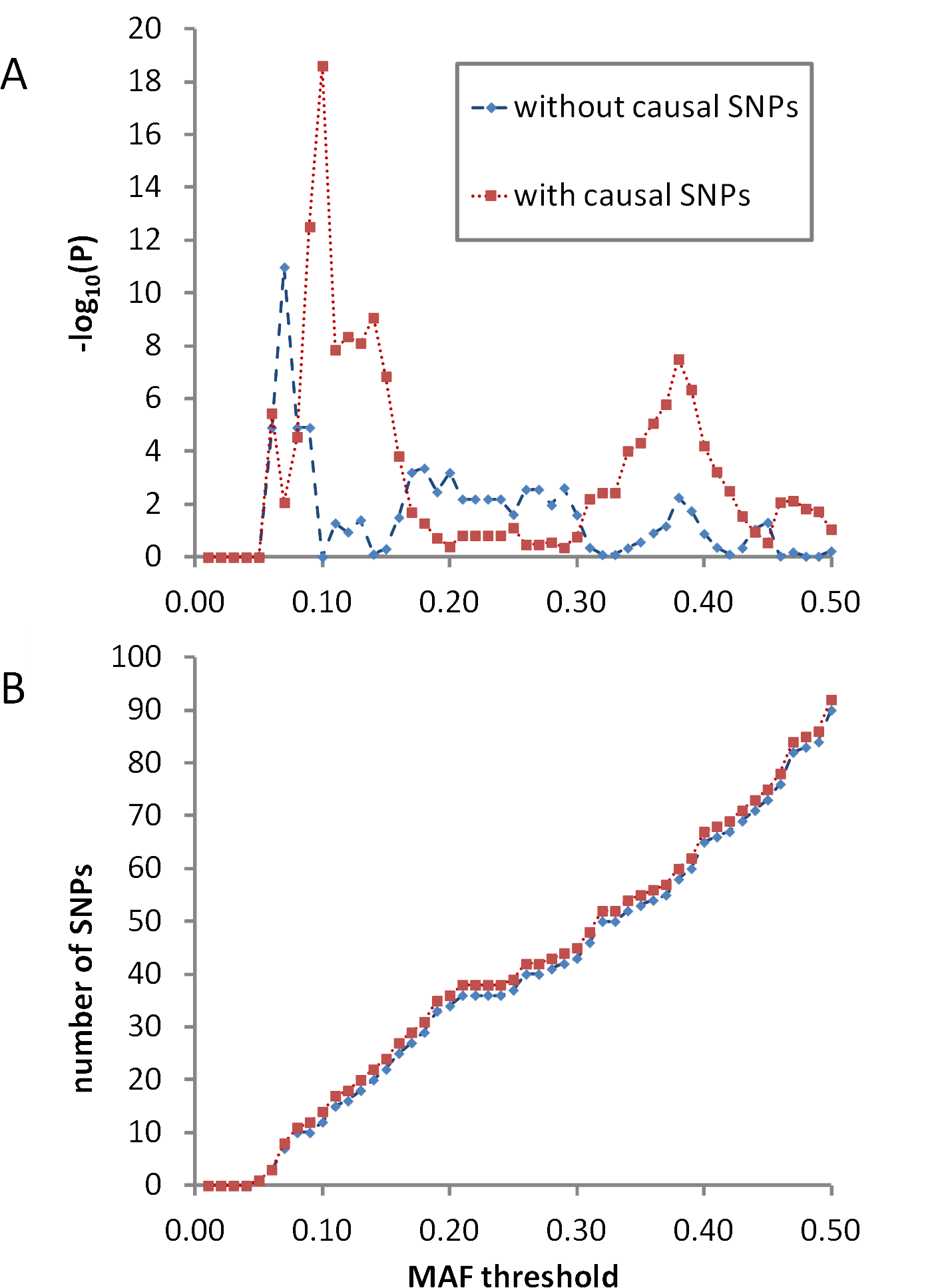

Supplement: Figure S5 — Association analysis of the MC1R SNPs and the red hair color using the weighted sum statistic (WSS). All number of genotyped SNPs in the 87.88 to 88.69 Mb region of (N SNPs = 90) were included to the WSS analysis according to the minor allele frequencies in the ascending order. The −log10(P) values from WSS were plotted against the MAF thresholds (blue dots). The analysis was then repeated by assuming that two causal SNPs rs1805007 and rs1805008 were available on the chip (red dots). A, the −log10(P) values; B, the number of SNPs included in the analysis. (TIF) [file pone.0028145.s005.tif]
