# Supplementary material for: Detecting Low Frequent Loss-of-Function Alleles in Genome Wide Association Studies with Red Hair Color as Example
Source: PLoS One. 2011 Nov 29;6(11):e28145. doi: 10.1371/journal.pone.0028145 (PMC3226656; doi:10.1371/journal.pone.0028145)
Supplement: Text S1 — Laboratory details for MC1R SNP genotyping. (DOC) [file pone.0028145.s006.doc]

**Text S1.** Laboratory details for MC1R SNP genotyping.

After optimization, 2 ng of dried DNA in clear 384-well plates (Applied Biosystems, Foster City, USA) was typed using a reaction volume of 5 µl. The reaction for SNP rs1805007 contains 1x LightCycler® 480 genotyping master (Roche, Mannheim, Germany), 0.5 µM forward primer, 1.0 µM reverse primer, 0.2 µM 3’-FL labeled probe and 0.2 µM 5’-LC labeled probe. PCR was performed in a Lightcycler® 480 (Roche) at 95^o^C for 10 minutes followed by 45 cycles amplification of 95^o^C for 10 seconds, 57^o^C for 10 seconds, 72^o^C for 15 seconds, followed by the melting curve from 40^o^C to 80^o^C. Because SNP rs1805008 proved to be problematic, due to many strong binding stem loops present in the area, Tib Molbiol designed an internal labeled primer which excludes some loops and breaks up the strongest inside of the amplification. This primer also functions as the sensor probe in the reaction. The reaction for SNP rs1805008 contains 1x LightCycler® 480 genotyping master (Roche), 0.25 µM forward primer, 0.5 µM reverse primer and 0.2 µM 3’-FL labeled probe. The amplification was performed in a Lightcycler® 480 (Roche) at 95^o^C for 10 minutes followed by 50 cycles amplification of 95^o^C for 10 seconds, 55^o^C for 10 seconds, 72^o^C for 10 seconds, followed by the melting curve from 40^o^C to 80^o^C.
