# Supplementary material for: Detecting Low Frequent Loss-of-Function Alleles in Genome Wide Association Studies with Red Hair Color as Example
Source: PLoS One. 2011 Nov 29;6(11):e28145. doi: 10.1371/journal.pone.0028145 (PMC3226656; doi:10.1371/journal.pone.0028145)
Supplement: Table S1 — Primers of two MC1R SNPs. (DOC) [file pone.0028145.s007.doc]

**Table S1.** Primers of two *MC1R* SNPs

| Primer/probe | rs1805007 | rs1805008 |
| --- | --- | --- |
| Forward primer | TGTCGGACCTGCTGGTGAG | CTCCATGCTGTCCAGCCTC |
| Reverse primer | ACGTGGTCGTAGTAGGCGATGA | CGCAACGGC XTCGACGC (internal labeled) |
| 5'-LC Labeled probe | 640-CACTGCGCTACCACAGC p |  |
| 3'-FL Labeled probe | TGGACCGCTACATCTCCATCTTCTAC-FL | GTGACCCTGCCGCGGGC-FL |
